# Supplementary material for: Locus specific epigenetic modalities of random allelic expression imbalance
Source: Nat Commun. 2021 Sep 9;12:5330. doi: 10.1038/s41467-021-25630-3 (PMC8429725; doi:10.1038/s41467-021-25630-3)
Supplement: Supplementary file 1 — Supplementary Information [file 41467_2021_25630_MOESM1_ESM.pdf]

Locus specific epigenetic modalities of random allelic expression imbalance

Lucile Marion-Poll, Benjamin Forêt, Dina Zielinski, Florian Massip, Mikael Attia, Ava C Carter, Laurène Syx, Howard Y Chang, Anne-Valerie Gendrel, Edith Heard

DESCRIPTION OF SUPPLEMENTARY INFORMATION:

Supplementary Figure 1: RT-qPCR of pluripotency markers and differentiation markers
Supplementary Figure 2: BIC criterion calculated for each gene for the Gaussian mixture models
Supplementary Figure 3: Schematic of the differentiation of NPC clones into populations of astrocytes
Supplementary Figure 4: Cumulative distributions for each of the six independent differentiation experiments
Supplementary Figure 5: Western blot analysis of BAG3 protein expression levels
Supplementary Figure 6: Local regulation of allelic imbalance

Supplementary Table 1: Number of NPC clones generated for each differentiation experiment
Supplementary Table 2: P-values of the likelihood ratio tests
Supplementary Table 3: Bootstrap estimates of the parameters
Supplementary Table 4: List of clones used for sequencing
Supplementary Table 5: List of compounds used in the screen
Supplementary Table 6: List of pyrosequencing primers
Supplementary Table 7: List of qPCR primers
Supplementary Table 8: List of bisulfite primers

SUPPLEMENTARY FIGURES

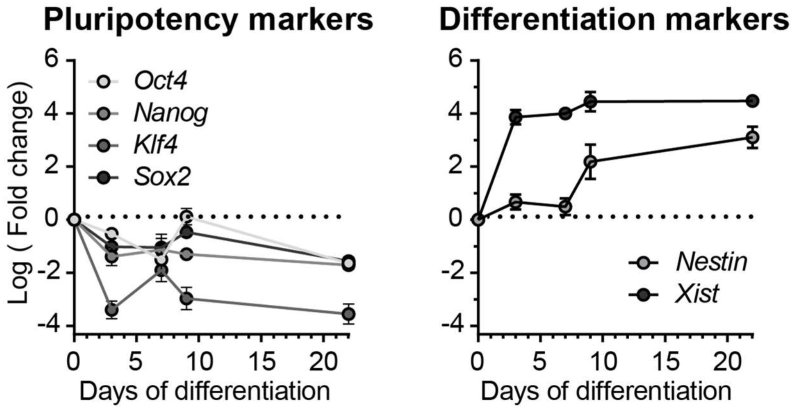

Supplementary Fig. 1
RT-qPCR of pluripotency markers and differentiation markers during the differentiation of ESCs towards NPCs. Data are means ± SEM (n = 4 replicates per condition).

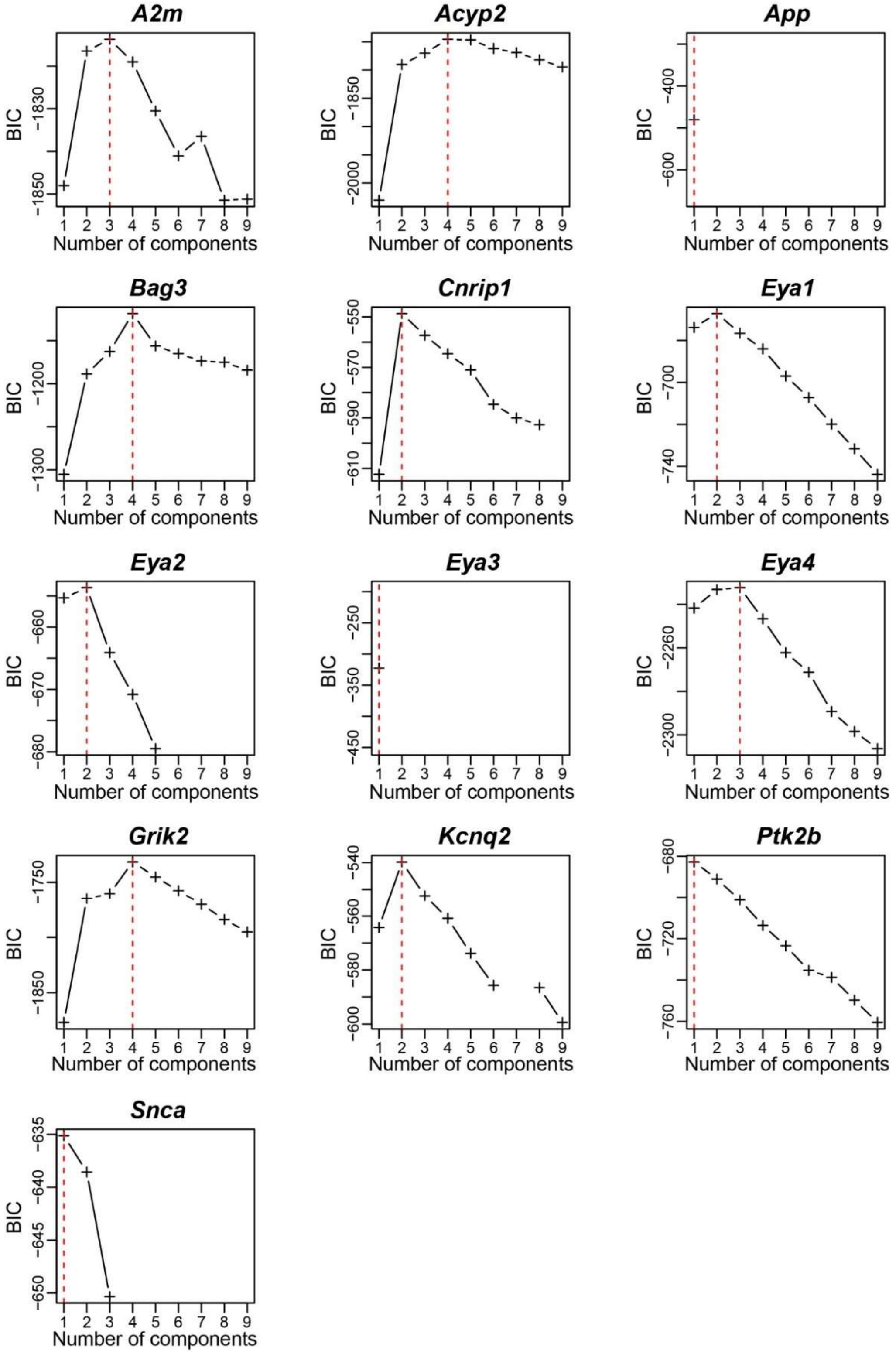

Supplementary Fig. 2
BIC criterion calculated for each gene for the Gaussian mixture models, with up to 9 components. The red dashed lines indicate the number of components maximizing the BIC criterion. For certain values of the number of components, the EM algorithm did not converge and therefore, the BIC could not be estimated (e.g., in the case of *Eya3* and *App*). This occured when the variance in the empirical data was too small and no good solution could be found.

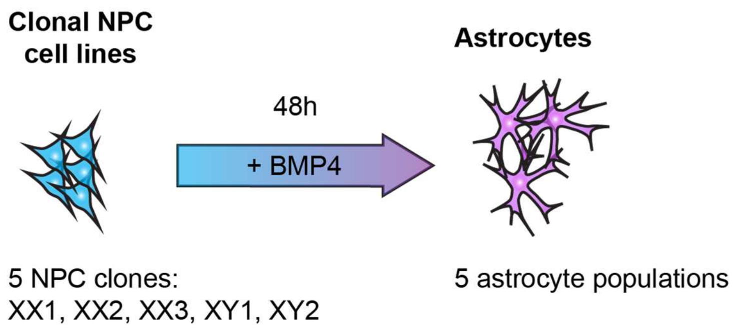

Supplementary Fig. 3
Experimental strategy to assess the stability of monoallelic expression after *in vitro* differentiation of 5 NPC clones into populations of astrocytes.

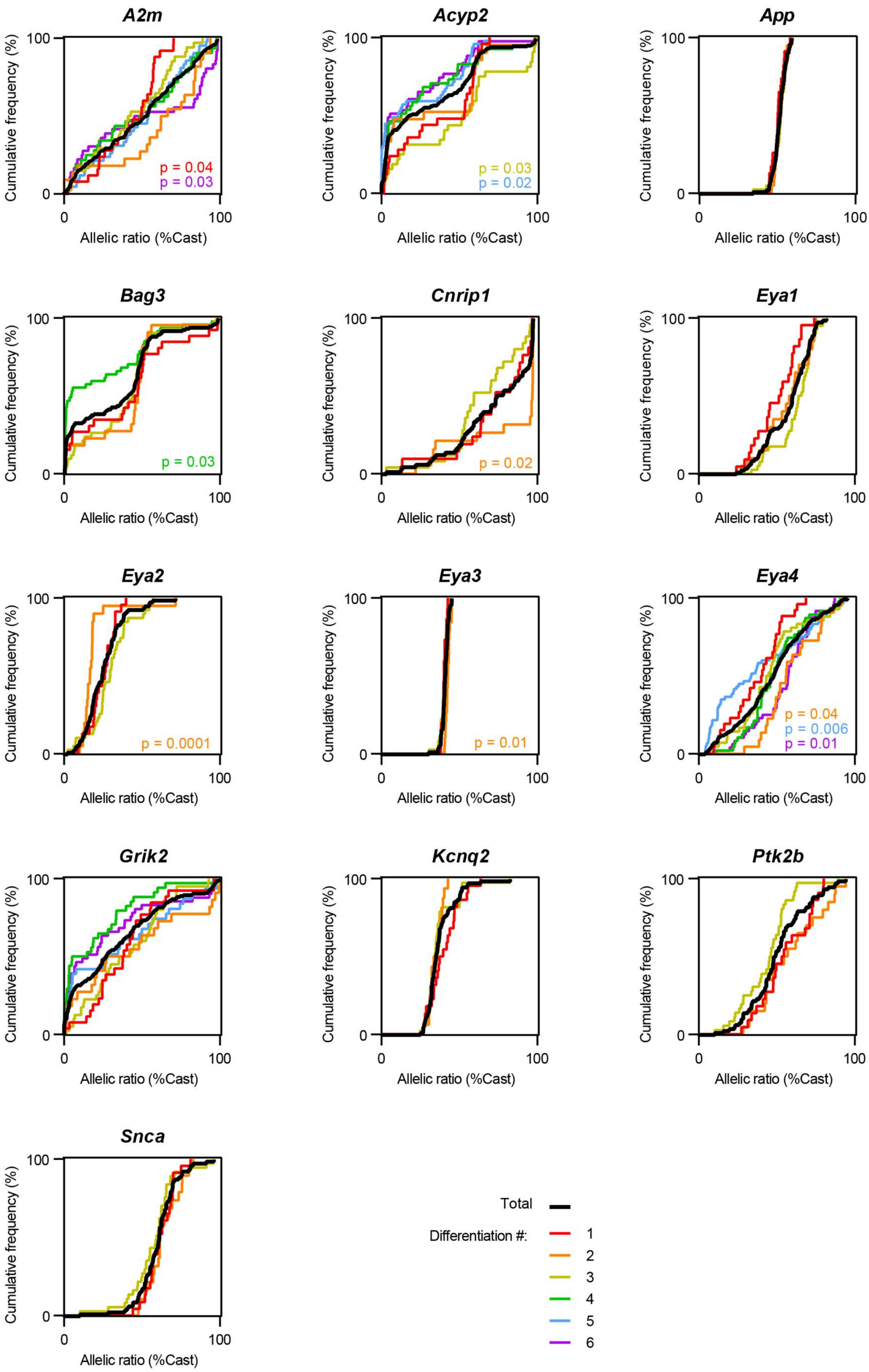

Supplementary Fig. 4

Cumulative distributions of allelic expression ratios for each gene of interest, in all NPC clones analysed (black lines), and each of the six independent differentiation experiments (colored lines). Kolmogorov-Smirnov tests, between the total cumulative distribution and the cumulative distribution associated with each experiment, without correction.

a

Lane	1	2	3	4	5	6	7	8	9	10	11
Clone	42	50	13	52	58	61	66	74	51	20	106
Allelic status	Mono	Bi	Bi	Bi	Mono	Mono	Mono	Mono	Bi	Mono	Bi
RNA level	High	High	High	High	Low	Low	High	High	Low	Low	Low

b

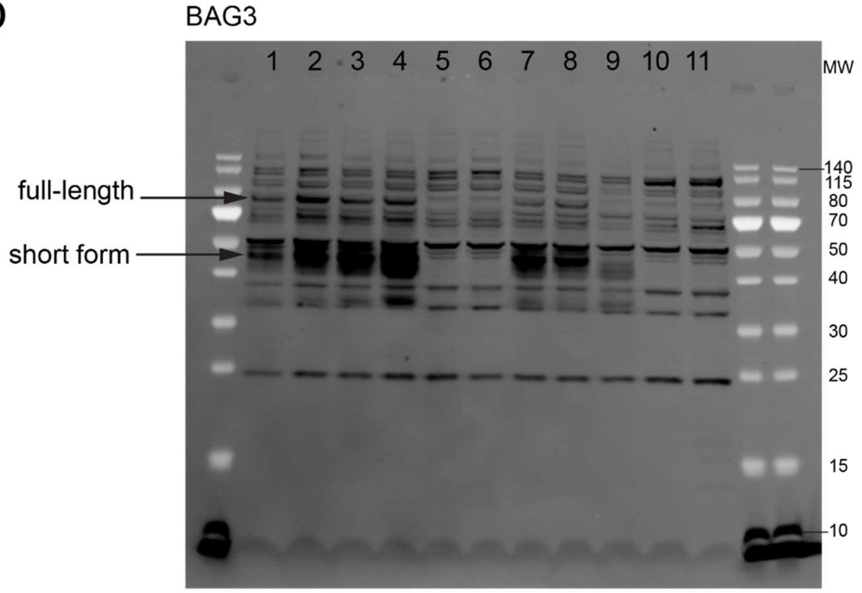

c

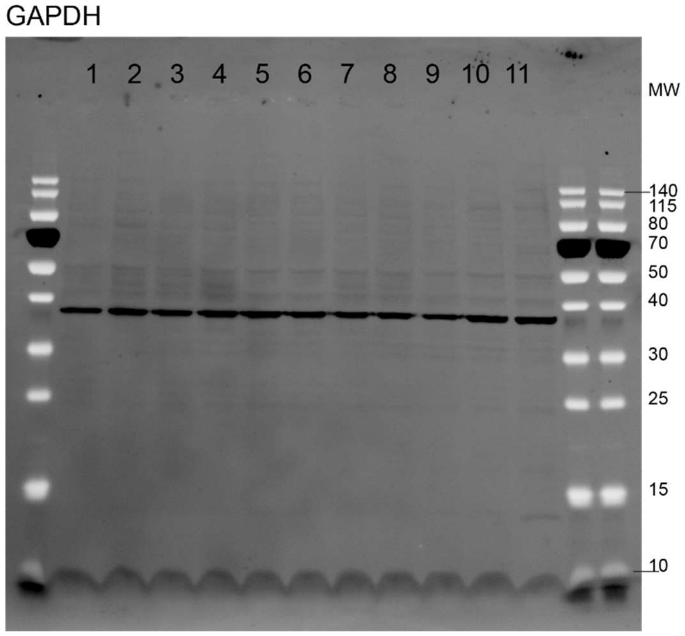

Supplementary Fig. 5
Western blot analysis of BAG3 protein expression levels. (a) Details of the 11 NPC clones analysed, characterized by high or low expression levels and mono- or biallelic expression. (**b**) Full image of the western blot with the BAG3 antibody. The full-lenght band was used for the quantification in Figure 5. (**c**) Full image of the western blot with the GAPDH antibody.

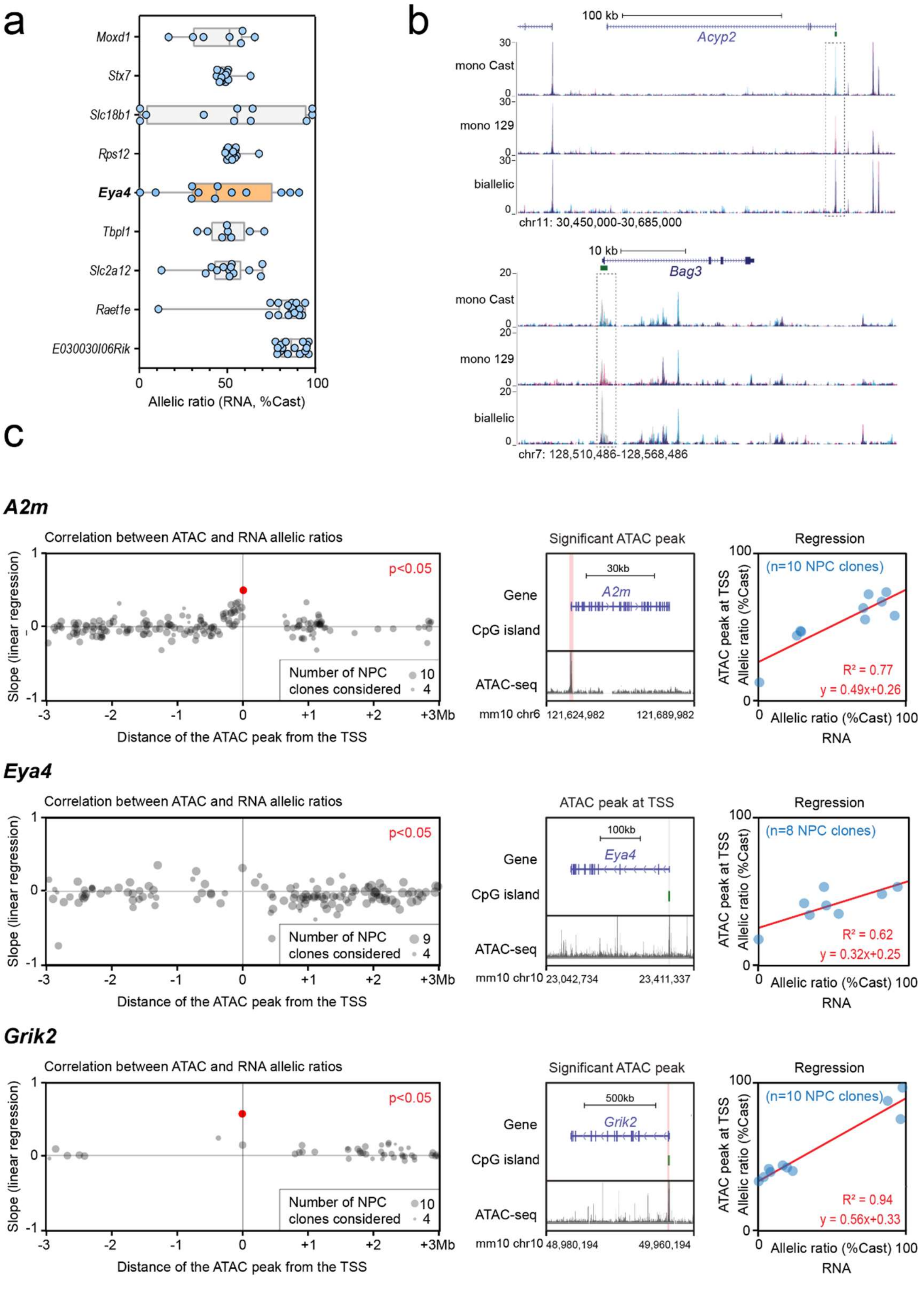

Supplementary Fig. 6
(**a**) Comparison of the distribution of allelic expression ratios for *Eya4*, with 4 upstream and 4 downstream neighbouring genes, measured by allele-specific RNA-seq in 16 NPC clones. Boxplot elements: center line represents the median, box bounds represent the 25th and 75th percentiles, whiskers represent the minimum and maximum. The orange color highlights the box plot of the RME gene of interest. (**b**) UCSC genome browser snapshots showing allele-specific ATAC-seq tracks for *Acyp2* and *Bag3* are shown for 3 representative clones (Cast-specific reads are shown in blue, 129-specific reads in pink, and non-allele specific reads in grey). Dashed grey boxes indicate the position of the random accessible monoallelic element for both genes. (**c**) Identification of the genomic region where allele-specific accessibility correlates best with expression for *A2m*, *Eya4* and *Grik2* by linear regression between the ATAC-seq allelic accessibility ratio and RNA-seq allelic expression ratio, for ATAC-seq peaks (containing a least a SNP) within a region of ±3 megabases around the TSS. *Pvalue* adjusted with Bonferroni correction, number of ATAC peaks: 176/128/47 *A2m*/*Eya4*/*Grik2* (left panels). Representative ATAC-seq track along the genes, with highlighting of the ATAC-seq peak, located at the TSS, that correlates significantly with the RNA-seq for *A2m* and *Grik2* (middle panels). Linear regression showing the correlation between the ATAC-seq allelic accessibility ratio and the RNA-seq allelic expression ratio at the TSS (right panels). ATAC-seq data and RNA-seq data from 13 NPC clones. *A2m* $F_{(1,8)} = 27$, $p = 8.5 \times 10^{-4}$; *Eya4* $F_{(1,6)} = 9.6$, $p = 0.021$; *Grik2* $F_{(1,8)} = 120$, $p = 4.3 \times 10^{-6}$

SUPPLEMENTARY TABLES

ES cell line	Differentiation n°	Number of NPC clones
F123 (male)	1	26
F123 (male)	2	22
F121.6 (female)	3	42
F121.6 (female)	4	48
F121.6 (female)	5	61
F121.6 (female)	6	50

Supplementary Table 1: Number of NPC clones generated for each differentiation experiment.

Gene	1 vs 2	2 vs 3	3 vs 4	4 vs 5
A2m Cast	0.001	0.003	0.032	
Acyp2 Cast	0.001	0.001	0.001	0.007
App Cast	NA			
Bag3 Cast	0.001	0.001	0.001	0.983
Cnrip1 Cast	0.001	0.368		
Eya1 Cast	0.002	0.312		
Eya2 Cast	0.007	0.366		
Eya3 Cast	NA			
Eya4 Cast	0.099	0.001	0.395	
Grik2 Cast	0.001	0.002	0.001	0.388
Kcnq2 Cast	0.001	0.822		
Ptk2b Cast	0.405			
Snca Cast	0.025			

Supplementary Table 2: P-values of the likelihood ratio tests.
P-values of the likelihood ratio test for each increment of the number of components. A small p-value indicates that likelihood improvement is large enough and is unlikely explained by noise in the data.

Gene	Component	Proportion	Mean	Variance
A2m Cast	1	17.1 (10.6-29.2)	6.08 (3.88 - 12.01)	12.66 (3.89 - 59.62)
A2m Cast	2	63.7 (39.8-74.2)	49.1 (34.9 - 57.1)	426 (192 - 515)
A2m Cast	3	18.23 (9.44-40.98)	88.1 (75.9 - 92.7)	44.1 (21.2 - 174.8)
Acyp2 Cast	1	37.5 (30.3-44.2)	2.41 (2.00 - 2.85)	2.34 (1.65 - 3.81)
Acyp2 Cast	2	27.7 (17.2-37.9)	23.9 (13.9 - 33.6)	191.7 (43.1 - 352.1)
Acyp2 Cast	3	29.3 (20.0-40.2)	58.1 (53.4 - 60.0)	27.6 (13.4 - 98.3)
Acyp2 Cast	4	5.67 (2.37-8.53)	97.0 (93.8 - 98.5)	4.525 (0.469 - 37.818)
App Cast	1	100 (100-100)	51.9 (50.9 - 52.7)	16.7 (11.2 - 25.3)
Bag3 Cast	1	22.2 (14.6-29.4)	0.899 (0.729 - 1.150)	0.1066 (0.0267 - 0.3103)
Bag3 Cast	2	56.1 (47.1-64.9)	47.2 (43.9 - 49.7)	105.9 (49.7 - 214.6)
Bag3 Cast	3	15.73 (8.93-24.02)	7.99 (3.62 - 13.54)	29.07 (1.27 - 79.04)
Bag3 Cast	4	5.84 (2.19-10.22)	97.1 (95.5 - 98.5)	3.856 (0.146 - 6.758)
Cnrip1 Cast	1	29.6 (18.0-41.6)	96.6 (96.1 - 97.0)	0.3999 (0.0562 - 1.0542)
Cnrip1 Cast	2	70.4 (58.4-82.0)	60.9 (54.1 - 67.0)	515 (322 - 731)
Eya1 Cast	1	69.0 (53.9-80.1)	65.5 (63.1 - 68.3)	50.4 (29.1 - 73.2)
Eya1 Cast	2	31.0 (19.9-46.1)	39.6 (36.0 - 45.2)	49.6 (22.5 - 114.0)
Eya2 Cast	1	7.3 (2.4-16.5)	55.5 (45.8 - 63.0)	50.973 (0.837 - 168.311)
Eya2 Cast	2	92.7 (83.5-97.6)	23.1 (20.7 - 25.1)	79.9 (56.6 - 107.5)
Eya3 Cast	1	100 (100-100)	40.8 (40.3 - 41.4)	4.85 (2.69 - 8.58)
Eya4 Cast	1	7.18 (3.47-14.06)	88.2 (82.9 - 91.0)	9.959 (0.797 - 43.568)
Eya4 Cast	2	82.3 (74.6-88.4)	48.0 (44.4 - 51.0)	308 (231 - 369)
Eya4 Cast	3	10.3 (5.8-15.6)	9.26 (7.12 - 11.60)	9.96 (2.59 - 22.07)
Grik2 Cast	1	33.3 (19.2-47.1)	52.4 (45.5 - 62.6)	196.4 (73.7 - 331.0)
Grik2 Cast	2	27.5 (14.1-42.7)	23.1 (17.9 - 29.4)	77.6 (13.3 - 151.2)
Grik2 Cast	3	30.0 (22.6-37.3)	2.53 (1.69 - 3.25)	4.46 (2.47 - 6.60)
Grik2 Cast	4	9.08 (5.07-13.37)	95.6 (94.4 - 96.8)	5.32 (2.35 - 9.29)
Kcnq2 Cast	1	72.7 (56.6-88.5)	33.5 (32.2 - 34.8)	13.78 (8.47 - 21.30)
Kcnq2 Cast	2	27.3 (11.5-43.4)	48.4 (44.4 - 54.2)	115.54 (5.63 - 260.01)
Ptk2b Cast	1	100 (100-100)	50.7 (47.0 - 54.8)	323 (234 - 425)
Snca Cast	1	100 (100-100)	60.4 (57.6 - 63.0)	155.5 (94.6 - 241.9)

Supplementary Table 3: Bootstrap estimates of the parameters.
Table presenting the estimates of the parameters (mean, variance and proportions of clones assigned to each population) for each gene and each component. Values in parenthesis give the 95% confidence intervals estimated via resampling (see Methods).

NPC clone/Astrocyte - RNAseq	RNAseq reference	RNAseq library ID (Curie)	NPC clone - ATACseq	ATACseq reference	Cell type
NPC_129 (NPC_XX-1)	Gendrel et al, 2014 (GSE54016)		NPC_XX2	Xu et al, 2017 (GSE84646)	NPC
NPC_C2 (NPC_XX-2)			NPC_XX1		NPC
NPC_G4 (NPC_XX-3)			NPC_XX4		NPC
NPC_E1.2 (NPC_XX-4)					NPC
NPC_mC3 (NPC_XY-1)			NPC_XY12	Xu et al, 2017 (GSE84646)	NPC
NPC_mE6 (NPC_XY-2)			NPC_XY14		NPC
NPC_mB4 (NPC_XY-3)			NPC_XY11		NPC
NPC_mG3 (NPC_XY-4)			NPC_XY15		NPC
NPC_DFT (NPC_D9B2)	Giorgetti et al, 2016				NPC
NPC_F5	This study (Curie platform)	B145T30	NPC_XX3	Xu et al, 2017 (GSE84646)	NPC
NPC_mG6		B145T32	NPC_XY16		NPC
NPC_mG2		B145T31			NPC
Ch3_1/Ch3_2	This study (Stanford)		NPC_XY7	Xu et al, 2017 (GSE84646)	NPC
Ch5_1/Ch5_2			NPC_XY8		NPC
Ch7_1/Ch7_2			NPC_XY9		NPC
Ch8_1/Ch8_2			NPC_XY10		NPC
Astro_129	This study (Curie platform)	A234T12			Astrocyte
Astro_mE6		A234T13			Astrocyte
Astro_mC3		A234T14			Astrocyte
Astro_C2		B129T1			Astrocyte
Astro_G4		B129T2			Astrocyte

Supplementary Table 4: List of clones used for sequencing.

(+)-JQ1	Decitabine	Mitomycin C	RGFP966
2-Methoxyestradiol (2-MeOE2)	Droxinostat	Mizoribine	Ricolinostat (ACY-1215)
3-deazaneplanocin A (DZNeP) HCl	EI1	MK-5108 (VX-689)	Roxadustat (FG-4592)
4SC-202	Ellagic acid	ML324	Rucaparib (AG-014699,PF-01367338) phosphate
A-366	ENMD-2076	MLN8054	Ruxolitinib (INCB018424)
Abexinostat (PCI-24781)	ENMD-2076 L-(+)-Tartaric acid	MM-102	RVX-208
AG-14361	Entacapone	Mocetinostat (MGCD0103)	Scriptaid
AG-490 (Tyrphostin B42)	Entinostat (MS-275)	Momelotinib (CYT387)	Selisistat (EX 527)
Alisertib (MLN8237)	EPZ004777	MS023	SGC 0946
AMG-900	EPZ011989	MS436	SGC707
Anacardic Acid	EPZ015666(GSK3235025)	Nedaplatin	SGC-CBP30
APTSTAT3-9R	Fedratinib (SAR302503, TG101348)	Nexturastat A	SGI-1027
AR-42	Filgotinib (GLPG0634)	Niraparib (MK-4827) tosylate	SGI-1776 free base
AT9283	Gandotinib (LY2784544)	Norfloxacin	Sirtinol
Aurora A Inhibitor I	Gemcitabine HCl	NVP-BSK805 2HCl	SMI-4a
AZ 960	Givinostat (ITF2357)	NVP-TNKS656	SNS-314 Mesylate
AZ6102	GSK J1	Oclacitinib	Sodium Phenylbutyrate
Azacitidine	GSK J4 HCl	OF-1	SP2509
AZD1208	GSK1070916	Ofloxacin	SRT1720 HCl
AZD1480	GSK1324726A (I-BET726)	OG-L002	S-Ruxolitinib (INCB018424)
AZD2461	GSK2801	Olaparib (AZD2281, Ku-0059436)	Tacedinaline (CI994)
Barasertib (AZD1152-HQPA)	GSK2879552 2HCl	ORY-1001 (RG-6016) 2HCl	TAK-901
Baricitinib (LY3009104, INCB028050)	GSK503	OTX015	TG101209
Belinostat (PXD101)	GSK591	Pacritinib (SB1518)	TMP269
BI-7273	GSK-LSD1 2HCl	Panobinostat (LBH589)	Tofacitinib (CP-690550) Citrate
BIX 01294	Hesperadin	PCI-34051	Tofacitinib (CP-690550,Tasocitinib)
Blasticidin S HCl	HLCL-61 HCL	PF-CBP1 HCl	Tozasertib (VX-680, MK-0457)
BRD4770	I-BET-762	PFI-1 (PF-6405761)	Tranylcypromine (2-PCPA) HCl
Bromosporine	I-BRD9	PFI-2 HCl	Trichostatin A (TSA)
C646	Iniparib (BSI-201)	PFI-3	Tubastatin A
Carboplatin	INO-1001 (3-Aminobenzamide)	PHA-680632	Tubastatin A HCl
CEP-33779	IOX1	Pinometostat (EPZ5676)	UNC0379
Clevudine	IOX2	Pirarubicin	UNC0631
CPI-169	ITSA-1 (ITSA1)	PJ34	UNC1215
CPI-203	JIB-04	PJ34 HCl	UNC669
CPI-360	JNJ-7706621	Pracinostat (SB939)	UPF 1069
CUDC-101	KW-2449	Procainamide HCl	Valproic acid sodium salt (Sodium valproate)
CUDC-907	Lomeguatrib	Procarbazine HCl	Veliparib (ABT-888)
CX-6258 HCl	M344	Quercetin	Vorinostat (SAHA, MK0683)
CYC116	MC1568	Quisinostat (JNJ-26481585) 2HCl	WHI-P154
Cytarabine	ME0328	Remodelin	WP1066
Dacinostat (LAQ824)	MG149	Resminostat	XL019
Danusertib (PHA-739358)	MI-2 (Menin-MLL Inhibitor)	Resveratrol	Zebularine
Daphnetin	MI-3 (Menin-MLL Inhibitor)	RG108	ZM 39923 HCl
Daptomycin	Mirin	RG2833 (RGFP109)	ZM 447439
Decernotinib (VX-509)			

Supplementary Table 5: List of compounds used in the screen.

Gene	Primer name	Modification	Primer sequence	Product size (bp)	SNP ID
A2m	A2M_F1	Biot	CAGAGCTCAGAAACCCAGAGTG	89	rs260691801
	A2M_R1		GGAGCACATAAGCGGTCATC		
	A2M_S1		GGTCATCTCTACCTCAGC		
Acyp2	Acyp2_pyro-F	Biot	ACACTTTCCATTTTTAACTCTCCT	105	rs48601327
	Acyp2_pyro-R		GTATTCGATATTAATGGAAGAAAA		
	Acyp2_pyro-S		AAAATAAAACATTCTTAGAT		
App	App_F1	Biot	CTGGTGTAGGAACTTGCACTTGT	96	rs48007435
	App_R1		TACGCGAATGATACAGGTCCA		
	App_S1		CGCCCTTCTCGTGCC		
Bag3	Bag3_pyro-F	Biot	TGCCCATCTCCCATCCGT	87	rs31544462
	Bag3_pyro-R		TTCGGGTTGGGTAACAGGTG		
	Bag3_pyro-S		GCTGAGGCCTGTCAAC		
Cnrip1	Cnrip1_pyro-F	Biot	TCAGTGGAAGTAGCAGGTTTGTC	129	rs26873596
	Cnrip1_pyro-R		GGGACTCCAGCACATTGTTATC		
	Cnrip1_pyro-S		TCTTGAAAACAAAACACC		
Eya1	Eya1_pyro-F		TGTTTCTGATGATGGCCAGACC	56	rs229395946
	Eya1_pyro-R	Biot	CACCCAGACAAGGTTCATTGC		
	Eya1_pyro-S		AGACCAACCCACCAT		
Eya2	Eya2_pyro-F		ATTTATGGACTGCACCCGTTACTC	75	rs220343389
	Eya2_pyro-R	Biot	AAACCAGACCTACAGCCGAGAA		
	Eya2_pyro-S		ACCCGTTACTCCCAT		
Eya3	Eya3_pyro-F	Biot	TCGGTGCTGTCCTGGAAGA	76	rs13466257
	Eya3_pyro-R		TACCTGGGGAGAGTGAGGAATG		
	Eya3_pyro-S		AGGAATGAGGCCCAA		
Eya4	Eya4_pyro-F	Biot	GCAGGCAATTGTTCAGCA	66	rs46855682
	Eya4_pyro-R		GGGTCTTCCCGTTACAGACA		
	Eya4_pyro-S		CCCGTTACAGACAGGA		
Grik2	Grik2_F1		AGGAAGGGAGGAACCCATGATAA	134	rs248603850
	Grik2_R1	Biot	CTCATGCCGCTGTGTCTATGAAC		rs2461479928
	Grik2_S1		TGTAACCCCGTGTAACT		
Kcnq2	Kcnq2_F1	Biot	TGTGAGCGTAGACTACCGATCCC	131	rs27642401
	Kcnq2_R1		TGGTTCCCAGGGCAATGTCT		
	Kcnq2_S1		GTGGCACCTGGAAGC		
Ptk2b	Ptk2b_F1		CCTCCGACTCTCTGCTTCTTCC	211	rs257352928
	Ptk2b_R1	Biot	AGGGGACAGAGAGGGGTTTTAT		
	Ptk2b_S1		TACACTGTACAAGAGAGC		
Snca	Snca_F1	Biot	GCTCCCTAGGCTTCTGAAGAACTC	117	rs225467378
	Snca_R1		AGCAGACGGCAGGAGACCA		
	Snca_S1		CTGCCCTTGCCTCTT		

Supplementary Table 6: List of pyrosequencing primers.

Gene	Primer name	Primer sequence	Tm (°C)	Product size (bp)
Acyp2	Acyp2_RT_F	CTGGTTGGCTGGGTGAAGAA	60.18	97
	Acyp2_RT_R	TGCTCAGCCAGGACTTCATG	60.04	
Bag3	Bag3_RT_F	AGCCGCCCAGACAGATAAAC	60.11	109
	Bag3_RT_R	GGCAGCTGGAGACTGAGATC	59.90	
Eya3	Eya3_RT_F	CCAGACGGAGAAGCCTAGTG	59.54	199
	Eya3_RT_R	CCGTTCCAATTCACTGTCCT	57.81	
Rrm2	Rrm2-RT_F	CCGAGCTGGAAAGTAAAGCG	59.28	91
	Rrm2-RT_R	ATGGGAAAGACAACGAAGCG	58.84	
B2m	B2m_F	ACCCGCCTCACATTGAAATCC	60.96	199
	B2m_R	CGATCCCAGTAGACGGTCTTG	59.93	
Gapdh	Gapdh_RT_F	TGACGTGCCGCCTGGAGAAAC	65.48	160
	Gapdh_RT_R	CCGGCATCGAAGGTGGAAGAG	63.08	

Supplementary Table 7: List of qPCR primers.

Gene	Primer name	Primer sequence	Ta	Product size (bp)	# of CpG
Acyp2	Acyp2_BS_F	AGGAAGAGAGAAATGGATGGTATAAGTTTTTTTT	71.0	406	27
	Acyp2_BS_R	CAGTAATACGACTCACTATAGGGAGAAGGCTAATTAATTTAATCCCCTCACATACC			
Bag3	Bag3_BS_F	AGGAAGAGAGGGTTTTAAGGTGAGTGGAGTTTGT	72.6	422	41
	Bag3_BS_R	CAGTAATACGACTCACTATAGGGAGAAGGCTACTCAACAACCTTTCAATCCTAAC			

Supplementary Table 8: List of bisulfite primers.
