# Supplementary material for: Locus specific epigenetic modalities of random allelic expression imbalance
Source: Nat Commun. 2021 Sep 9;12:5330. doi: 10.1038/s41467-021-25630-3 (PMC8429725; doi:10.1038/s41467-021-25630-3)
Supplement: Supplementary file 2 — Reporting Summary [file 41467_2021_25630_MOESM2_ESM.pdf]

nature research

Corresponding author(s): Lucile Marion-Poll, Anne-Valerie Gendrel, Edith Heard

Last updated by author(s): Jul 23, 2021

Reporting Summary

Nature Research wishes to improve the reproducibility of the work that we publish. This form provides structure for consistency and transparency in reporting. For further information on Nature Research policies, see our Editorial Policies and the Editorial Policy Checklist.

Statistics

For all statistical analyses, confirm that the following items are present in the figure legend, table legend, main text, or Methods section.

n/a	Confirmed	
☐	☒	The exact sample size (n) for each experimental group/condition, given as a discrete number and unit of measurement
☐	☒	A statement on whether measurements were taken from distinct samples or whether the same sample was measured repeatedly
☐	☒	The statistical test(s) used AND whether they are one- or two-sided<br>*Only common tests should be described solely by name; describe more complex techniques in the Methods section.*
☐	☒	A description of all covariates tested
☐	☒	A description of any assumptions or corrections, such as tests of normality and adjustment for multiple comparisons
☐	☒	A full description of the statistical parameters including central tendency (e.g. means) or other basic estimates (e.g. regression coefficient) AND variation (e.g. standard deviation) or associated estimates of uncertainty (e.g. confidence intervals)
☐	☒	For null hypothesis testing, the test statistic (e.g. F, t, r) with confidence intervals, effect sizes, degrees of freedom and P value noted<br>*Give P values as exact values whenever suitable.*
☐	☒	For Bayesian analysis, information on the choice of priors and Markov chain Monte Carlo settings
☒	☐	For hierarchical and complex designs, identification of the appropriate level for tests and full reporting of outcomes
☐	☒	Estimates of effect sizes (e.g. Cohen's d, Pearson's r), indicating how they were calculated

Our web collection on statistics for biologists contains articles on many of the points above.

Software and code

Policy information about availability of computer code

Data collection	Metamorph (microscopy image acquisition), ChemDoc MP Imaging system Bio-Rad (Western Blot)
Data analysis	ImageJ 1.52i (image analysis), PyroMark Assay Design software 2.0 & PyroMark Q24 Qiagen (pyrosequencing), XLSTAT Addinsoft (heatmap and Gaussian mixture model), SNPsplit 0.3.2, Tophat 2.1.0, Bowtie2 2-2.2.5, FeatureCounts 1.5.1, mclust 5.4.7, UCSC genome browser mm10 (sequencing data visualization)

For manuscripts utilizing custom algorithms or software that are central to the research but not yet described in published literature, software must be made available to editors and reviewers. We strongly encourage code deposition in a community repository (e.g. GitHub). See the Nature Research guidelines for submitting code & software for further information.

Data

Policy information about availability of data

All manuscripts must include a data availability statement. This statement should provide the following information, where applicable:

- Accession codes, unique identifiers, or web links for publicly available datasets
- A list of figures that have associated raw data
- A description of any restrictions on data availability

Raw (FASTQ files) and processed RNAseq data generated in this study were deposited in GEO under accession number GSE148348.
Previously published sequencing data used in this study are available in the GEO database under accession codes GSE54016, GSE72697 and GSE84646.

Field-specific reporting

Please select the one below that is the best fit for your research. If you are not sure, read the appropriate sections before making your selection.

☒ Life sciences ☐ Behavioural & social sciences ☐ Ecological, evolutionary & environmental sciences

For a reference copy of the document with all sections, see nature.com/documents/nr-reporting-summary-flat.pdf

Life sciences study design

All studies must disclose on these points even when the disclosure is negative.

Sample size	Sample sizes were determined based on our previous and similar experiments. The assessment of the allelic ratio distributions was novel and the sample size could not be predetermined, thus we completed the n for the most variable genes after a first series of experiments.
Data exclusions	No data were excluded from the analysis
Replication	We performed 6 independent differentiation experiments of ES cells into NPC, which led to consistent results. For the western blot, all the clones analysed are shown in the manuscript. For the RNA-FISH, consistent results were obtained from all 3 animals. The effect of the epidrug screen hits was confirmed in 4 independent experiments, ie for all attempts at replication.
Randomization	For the epidrug screen, the treated and untreated cells originated from the same flasks that were split. Randomization does not apply to other experiments as we did not apply different treatments.
Blinding	Blinding was not applicable for microscopy experiments, as there was only one condition. For all the other experiments, the experimenter was unaware of the previous results concerning each individual clone when collecting the data (ie blind to the allelic category and expression levels).

Reporting for specific materials, systems and methods

We require information from authors about some types of materials, experimental systems and methods used in many studies. Here, indicate whether each material, system or method listed is relevant to your study. If you are not sure if a list item applies to your research, read the appropriate section before selecting a response.

Materials & experimental systems

n/a	Involved in the study	
☐	☒	Antibodies
☐	☒	Eukaryotic cell lines
☒	☐	Palaeontology and archaeology
☐	☒	Animals and other organisms
☒	☐	Human research participants
☒	☐	Clinical data
☒	☐	Dual use research of concern

Methods

n/a	Involved in the study	
☒	☐	ChIP-seq
☒	☐	Flow cytometry
☒	☐	MRI-based neuroimaging

Antibodies

Antibodies used	BAG3 (rabbit polyclonal, 10599-1-AP, Proteintech, batch 00053771) , GAPDH (mouse monoclonal, ab9484, Abcam), anti-rabbit conjugated with Alexa Fluor 488 (Invitrogen, A-11034), anti-mouse conjugated with Alexa Fluor 546 (Invitrogen, A-11030)
Validation	The BAG3 antibody has been validated for WB on the manufacturer's website "KD/KO validated", and cited in 71 publications. The GAPDH antibody has been validated by Abcam for WB, and has over 600 citations.

Eukaryotic cell lines

Policy information about cell lines

Cell line source(s)	The female F1-21.6 and male F1-23 mouse ESC lines were a kind gift from Prof. Joost Gribnau
Authentication	No specific authentication procedure was performed
Mycoplasma contamination	All cell lines were tested negative for mycoplasma
Commonly misidentified lines (See ICLAC register)	None used in this study

Animals and other organisms

Policy information about studies involving animals; ARRIVE guidelines recommended for reporting animal research

Laboratory animals	The experiments were conducted with 15-week old female C57Bl/6J mice (Charles River, France). The animals were housed in group, in a 12-hour light–dark cycle, in stable conditions of temperature, with food and water ad libitum.
Wild animals	The study did not involve wild animals
Field-collected samples	The study did not involve any sample collected from the field
Ethics oversight	Experiments were in accordance with the European Community Council Directive 2010/63/EU and approved by the ethics committee of the Institut Curie CEEA-IC#118 and authorized by the Ministère de l'Education Nationale, de l'Enseignement Supérieur et de la Recherche (APAFIS#8812-2017020611033784 v2).

Note that full information on the approval of the study protocol must also be provided in the manuscript.
